# Scale Effects in Orthotropic Composite Assemblies as Micropolar Continua: A Comparison between Weak- and Strong-Form Finite Element Solutions

**DOI:** 10.3390/ma12050758

**Published:** 2019-03-05

**Authors:** Lorenzo Leonetti, Nicholas Fantuzzi, Patrizia Trovalusci, Francesco Tornabene

**Affiliations:** 1 DINCI-Department of Civil Engineering, University of Calabria, 87036 Rende, Italy; lorenzo.leonetti@unical.it; 2 DICAM-Department, School of Engineering and Architecture, University of Bologna, 40136 Bologna, Italy; nicholas.fantuzzi@unibo.it; 3 DISG-Department of Structural and Geotechnical Engineering, Sapienza University of Rome, 00185 Rome, Italy; 4 DII-Department of Innovation Engineering, University of Salento, 73100 Lecce, Italy; francesco.tornabene@unisalento.it

**Keywords:** cosserat continua, orthotropic composites, brick/block masonry, finite element method, differential quadrature method, strong-formulation finite element method

## Abstract

The aim of the present work was to investigate the mechanical behavior of orthotropic composites, such as masonry assemblies, subjected to localized loads described as micropolar materials. Micropolar models are known to be effective in modeling the actual behavior of microstructured solids in the presence of localized loads or geometrical discontinuities. This is due to the introduction of an additional degree of freedom (the micro-rotation) in the kinematic model, if compared to the classical continuum and the related strain and stress measures. In particular, it was shown in the literature that brick/block masonry can be satisfactorily modeled as a micropolar continuum, and here it is assumed as a reference orthotropic composite material. The in-plane elastic response of panels made of orthotropic arrangements of bricks of different sizes is analyzed herein. Numerical simulations are provided by comparing weak and strong finite element formulations. The scale effect is investigated, as well as the significant role played by the relative rotation, which is a peculiar strain measure of micropolar continua related to the non-symmetry of strain and work-conjugated stress. In particular, the anisotropic effects accounting for the micropolar moduli, related to the variation of microstructure internal sizes, are highlighted.

## 1. Introduction

Complex composite materials are characterized by the presence of a heterogeneous and discontinuous internal structure, if observed at some length scales. A basic issue of mechanics of complex materials is the derivation of suitable constitutive models accounting for the presence of such internal structures (conventionally referred to as microstructures), as well as of its own evolution, due to plasticity, damage, fracture, contact with or without friction, and other nonlinear phenomena [1,2,3,4,5,6,7,8].

Discrete approaches, relying on direct modeling at smaller scales, were extensively used in the literature for simulating the mechanical behavior of composite materials, as well as other heterogeneous materials, including polycrystalline materials, jointed rock systems, and block masonry with periodic microstructures [9,10,11,12,13,14]. Such approaches, although numerically accurate in predicting the mechanical behavior of complex materials, are characterized by a huge computational cost, especially in the case of large-sized systems, for which continuum models are preferred.

Nevertheless, it is well known that the adoption of a classical and local (i.e., Cauchy of grade 1) continuum is often unsatisfactory to represent the real behavior of microstructured materials, especially when such materials are made of particles of significant size characterized by various anisotropic dispositions and orientations. For these systems, various enhanced non-classical continuous models, including micropolar (Cosserat), second-gradient, strain-rate, and continua with configurational forces [15,16,17,18], which exploit the advantages of a coarse-scale field description while keeping the memory of the fine organization of the material, were largely and satisfactorily adopted in the context of multiscale/multifield modeling [19,20,21,22,23,24,25,26,27,28,29,30,31,32,33,34,35,36,37]. These models can be defined as non-local due to the presence of both internal length scales and dispersion properties, revealing the existence of an underlying microstructure which inevitably affects the macroscopic mechanical properties [34].

Among these models, which were widely proven to be able to account for the usually experienced scale effects, attention is here focused on the micropolar model. This model was investigated for a long time from both theoretical and experimental points of view [38,39,40,41,42,43,44,45,46,47]. This continuum was preferred over the classical Cauchy continuum in many micromechanical approaches addressed to derive the macroscopic mechanical properties of masonry in both linear and nonlinear regimes [20,23,25,26]. This choice is particularly useful in the presence of nonlinear softening behaviors, due to the regularization properties of Cosserat models [41]. Moreover, several studies showed the efficacy of micropolar theories for solving practical problems in material and structural engineering. In particular, the Cosserat model was demonstrated to be equivalent to an assembly of rigid particles undergoing homogeneous displacements and rotations and interacting with each other via forces and couples; therefore, it is suitable for simulating the mechanical response of granular and masonry-like materials with either periodic or random microstructure [20,21,23,25,26,32,33,36].

Many explicit solutions for isotropic Cosserat materials were derived over the years [39,40], but the rather frequent case of anisotropic materials (typically encountered in masonry and other brick/block systems) often requires the use of numerical models and solution methods to predict their structural response. It is worth noting that, in Reference [20], it was shown that the anisotropic Cosserat continuum tends to behave as a Cauchy continuum, as the internal characteristic length goes to zero only when the material is at least orthotetragonal (e.g., in the unrealistic case of masonry made of square blocks with no-interlocking). This implies that it is not generally possible to describe an orthotropic medium as a Cauchy continuum, even if the microstructure is made of particles of vanishing size, as occurs in the well-known isotropic case. Furthermore, in the case of orthotropic materials, the relative rotations, implying non-symmetric angular strain components and work-conjugate non-symmetric stress components, play an important role which cannot be properly accounted for by adopting other generalized continua, such as second-gradient or couple-stress ones, as shown in References [32,36].

In the present work, the mechanical behavior of two-dimensional (2D) composite block assemblies modeled as orthotropic Cosserat continua is analyzed, devoting special attention to the material texture and the scale effects. By analyzing the response of brick/block assemblies of different size, and in particular by focusing on the effect of the relative rotation, the role of the induced anisotropy given by the material internal length is investigated. The investigation involves the variation of the overall bending moduli within a range with a reasonable physical meaning.

Finally, for the sake of comparison and to select the most appropriate numerical approach for the solution of the micropolar elastic problem, discussed in detail in Reference [48] with reference to a heterogeneous elastic panel in tension, two different numerical approaches based on weak and strong formulations are adopted herein. The results provided by the finite element method (FEM) are carried out using an in-house finite element formulation in terms of mixed bi-quadratic displacement and bi-linear micro-rotations implemented in COMSOL Multiphysics®, and the so-called strong-formulation finite element method (SFEM) [49,50,51,52,53,54]. The results are presented in terms of contour plots for both displacements and stresses. The main advantages and disadvantages of both methods in terms of convergence, stability and reliability, accuracy, and computational cost are critically discussed [55,56]. Summarizing, this work is structured as follows: Section 2 presents the micropolar continuum formulation for anisotropic solids. Section 3 gives the numerical FEM and SFEM formulations which implement the present micropolar continuum. Section 4 illustrates the numerical applications of a 2D planar micropolar solid subjected to uniform and concentrated loads with different material configurations. Finally, conclusions and remarks are given in Section 5.

## 2. The Micropolar Continuum Formulation for Anisotropic Solids

The micropolar (i.e., Cosserat) continuum was investigated for a long time from theoretical, numerical, and experimental points of view [40,41,42,43,44,45,46,47]. Eringen [15] formulated the micropolar model as a special case of micromorphic continua, which are characterized by microscopic deformation modes in addition to classical (macroscopic) ones. In particular, this continuum is made of material particles described by not only their position (as in Cauchy continua) but also their orientation. The kinematical descriptors of this model are displacements (macro-displacements) and rotations (micro-rotations), represented by the components $u_{i}$ and $\phi_{i}$ (with i=1,2,3), respectively. Coherently, each material particle possesses six degrees of freedom.

Within the framework of linearized kinematics, the following compatibility equations hold:

(1) $$\varepsilon_{ij}=u_{i,j}+e_{ijk}\phi_{k},\;\chi_{ij}=\phi_{i,j},$$

where $\varepsilon_{ij}$ and $\chi_{ij}$ denote the (non-symmetric) strain and curvature tensors, respectively, and $e_{ijk}$ is the usual third-order permutation tensor.

It follows that two stress measures work-conjugated to $\varepsilon_{ij}$ and $\chi_{ij}$ must be considered in the balance equations, i.e., the non-symmetric stress and couple-stress tensors, denoted by $\sigma_{ij}$ and $\mu_{ij}$, respectively. Under the simplifying hypothesis of neglected body couples, the balance equations to be satisfied for each point of the micropolar body are

(2) $$\sigma_{ij,j}+f_{i}=0,\;\mu_{kj,j}-e_{ijk}\sigma_{ij}=0,$$

where $f_{i}$ denotes the body forces, while body couples are considered null. Moreover, from equilibrium considerations at the external boundary, the surface tractions $t_{i}$ and moment tractions $m_{i}$ are expressed in terms of $\sigma_{ij}$ and $\mu_{ij}$ as $t_{i}=\sigma_{ij}n_{j}$ and $m_{i}=\mu_{ij}n_{j}$, respectively.

The general linear anisotropic stress–strain relations of the micropolar continuum can be expressed as

(3) $$\sigma_{ij}=A_{ijkl}\varepsilon_{kl}+B_{ijkl}\chi_{kl},\;\mu_{ij}=C_{ijkl}\varepsilon_{kl}+D_{ijkl}\chi_{kl}.$$

The total number of coefficients in Equation (3) is equal to 324; however, owing to the major symmetry requirements related to the existence of a well-defined strain energy function, the number of independent coefficients reduces to 171. Specific material symmetries imply further reduction of the number of elastic constants, and the representation of the above constitutive law can be simplified based on the symmetry properties of the considered microstructure. It can be shown for instance that, for centrosymmetric materials, the fourth-order tensors $B_{ijkl}$ and $C_{ijkl}$ traducing coupling between classical and curvature deformation effects vanish.

In the remainder of the paper, the attention is restricted to the case of a two-dimensional (2D) reduced model. Thus, each material particle of the continuum has only three degrees of freedom (DOFs), consisting in two in-plane displacement components, i.e., $u_{1}$ and $u_{2}$, and one out-of-plane micro-rotation component, i.e., $\phi_{3}$. Coherently, the out-of-plane strain and stress components, as well as the in-plane curvature and couple-stress components, are not considered in the stress–strain relations, which assume the following matrix form:

(4) $$\left[\begin{array}{c} \sigma_{11}\\ \sigma_{22}\\ \sigma_{12}\\ \sigma_{21}\\ \mu_{31}\\ \mu_{32} \end{array}\right]=\left[\begin{array}{cccccc} A_{1111} & A_{1122} & A_{1112} & A_{1121} & B_{111} & B_{112}\\ A_{2211} & A_{2222} & A_{2212} & A_{2221} & B_{221} & B_{222}\\ A_{1211} & A_{1222} & A_{1212} & A_{1221} & B_{121} & B_{122}\\ A_{2111} & A_{2122} & A_{2112} & A_{2121} & B_{211} & B_{212}\\ C_{111} & C_{122} & C_{112} & C_{121} & D_{11} & D_{12}\\ C_{211} & C_{222} & C_{212} & C_{221} & D_{21} & D_{22} \end{array}\right]\left[\begin{array}{c} \varepsilon_{11}\\ \varepsilon_{22}\\ \varepsilon_{12}\\ \varepsilon_{21}\\ \chi_{31}\\ \chi_{32} \end{array}\right].$$

In the considered case of hyperelastic materials having major symmetries, the following relations hold:

(5) $$A_{ijkl}=A_{klij},\;B_{ijk}=C_{kij},\;D_{ij}=D_{ji},$$

and the resulting number of independent elastic constitutive parameters turns to be 21. For centrosymmetric materials $B_{ijk}=C_{kij}=0$, the number of independent constitutive components is reduced to 13. Moreover, for orthotropic materials, which is the case of common running bond masonries, the additional conditions $A_{1112}=A_{1121}=A_{2212}=A_{2221}=0$ and $D_{12}=0$ hold, and the number of material constants becomes 8.

## 3. Numerical Formulations for Anisotropic Micropolar Models

In this section, the theoretical formulations for the two numerical approaches employed for comparison purposes in the present work, i.e., the (classical) finite element and the strong finite element methods, are discussed. In particular, a non-standard anisotropic micropolar finite element was formulated and subsequently implemented within the finite element environment COMSOL Multiphysics^®^, whereas the strong-form finite element method (SFEM) was implemented, for the same continuum model, via an in-house code written in MATLAB^®^. In this section, the main implementation details of the above formulations are provided.

### 3.1. Finite Element Formulation

The governing equations of the micropolar linear elasticity problem in a 2D setting can be discretized via a standard displacement-based finite element approach, after introducing the following vectors collecting the relevant displacements,

(6) $$u=\left[\begin{array}{c} u_{1}\\ u_{2} \end{array}\right],\;\phi =\left[\phi_{3}\right],$$

and strain components in the 2D framework:

(7) $$\varepsilon =\left[\begin{array}{c} \varepsilon_{11}\\ \varepsilon_{22}\\ \varepsilon_{12}\\ \varepsilon_{21} \end{array}\right],\;\chi =\left[\begin{array}{c} \chi_{31}\\ \chi_{32} \end{array}\right].$$

Similarly, the corresponding stress and couple stress vectors are introduced as follows:

(8) $$\sigma =\left[\begin{array}{c} \sigma_{11}\\ \sigma_{22}\\ \sigma_{12}\\ \sigma_{21} \end{array}\right],\;\mu =\left[\begin{array}{c} \mu_{31}\\ \mu_{32} \end{array}\right].$$

Via standard variational arguments, the boundary value problem defined in Section 2 can be reformulated in weak form, which is based on the virtual work principle for a 2D anisotropic micropolar continuum under the assumption of zero body couples and considering u and ϕ as a set of kinematically admissible displacement and rotation fields, respectively, such that

(9) $$\int_{\Omega }\delta \varepsilon^{T}\sigma d\Omega +\int_{\Omega }\delta \chi^{T}\mu d\Omega =\int_{\Omega }\delta u^{T}fd\Omega +\int_{\Gamma_{N}}\delta u^{T}\bar{t}d\Gamma +\int_{\Gamma_{N}}\delta \phi^{T}\bar{m}d\Gamma \;\forall \delta u,\delta \phi ,$$

where δ denotes the variational operator, f is the body force vector, and $\bar{t}$ and $\bar{m}$ are the traction and couple-traction vectors applied on the boundary $\Gamma_{N}$. It is useful to note that the curvature vector χ contains the first-order partial derivatives of the micro-rotations. This means that the strong continuity requirement is not necessary and $C^{0}$ shape functions can be adopted for the dependent kinematic variables u and ϕ.

It follows that the components of dependent variables u and ϕ in Equation (6) can be interpolated at any point in terms of their nodal values $\tilde{u}$ and $\tilde{\phi }$, referred to as primal unknowns of the present finite element formulation as

(10) $$u=N_{u}\tilde{u},\;\phi =N_{\phi }\tilde{\phi },$$

where $N_{u}$ and $N_{\phi }$ are the shape function matrices for u and ϕ. It is worth noting that different shape functions for displacements and micro-rotations are used, as shown in Reference [55]. Coherently, different Cosserat finite element formulations can be derived, by simply changing the order of these shape functions. In the present work, nine-node quadrangular elements were used, characterized by bi-quadratic and bi-linear interpolation functions for displacements and micro-rotations, respectively. It follows that all nine nodes possess displacement-type DOFs, whereas micro-rotation DOFs are referred only to the four corner nodes. The above shape function matrices can be collectively represented in the following matrix forms:

(11) $$\begin{array}{l} N_{u}=\left[\begin{array}{cc} \begin{array}{cc} N_{1}^{u} & 0\\ 0 & N_{1}^{u} \end{array} & \begin{array}{cc} \cdots & \begin{array}{cc} N_{9}^{u} & 0\\ 0 & N_{9}^{u} \end{array} \end{array} \end{array}\right],\\ N_{\phi }=\left[\begin{array}{ccc} N_{1}^{\phi } & \cdots & N_{4}^{\phi } \end{array}\right], \end{array}$$

where $N_{j}^{u}\left(\xi_{1},\xi_{2}\right)$ and $N_{j}^{\phi }\left(\xi_{1},\xi_{2}\right)$ are the biquadratic and bilinear shape functions for the jth node, respectively. It is worth noting that these functions are expressed in terms of natural coordinates $-1\leq \xi_{i}\leq 1$.

Consequently, the micropolar strains defined in Equation (1) can be expressed in the following matrix form:

(12) $$\begin{array}{l} \varepsilon =Lu+M\phi \;\mathrm{with}\;L={\left[\begin{array}{rrrr} \partial /\partial x_{1} & 0 & \partial /\partial x_{2} & 0\\ 0 & \partial /\partial x_{2} & 0 & \partial /\partial x_{1} \end{array}\right]}^{T},\;M={\left[\begin{array}{cccc} 0 & 0 & 1 & -1 \end{array}\right]}^{T},\\ \chi =\nabla \phi , \end{array}$$

where ∇ is the gradient operator in the 2D setting. If Equation (10) is substituted into Equation (12), the strain and curvature matrices can be expressed as

(13) $$\begin{array}{l} \varepsilon =\left[\begin{array}{cc} LN_{u} & MN_{\phi } \end{array}\right]\left\{\begin{array}{c} \tilde{u}\\ \tilde{\phi } \end{array}\right\}=B_{\varepsilon }d,\\ \chi =\left[\begin{array}{cc} 0 & \nabla N_{\phi } \end{array}\right]\left\{\begin{array}{c} \tilde{u}\\ \tilde{\phi } \end{array}\right\}=B_{\chi }d, \end{array}$$

where $d={\left[\begin{array}{cc} u^{T} & \phi^{T} \end{array}\right]}^{T}$ is the unknown vector of nodal (generalized) displacements. The matrices $B_{\varepsilon }$ and $B_{\chi }$ collect the spatial derivatives of the shape functions $N_{u}$ and $N_{\phi }$. It is worth noting that the computation of the inverse of Jacobian matrix is required to evaluate these shape functions, since they are functions of natural coordinates. Therefore, the constitutive relations in Equation (4) can be expressed in the following compact form:

(14) $$\sigma =D_{\varepsilon \varepsilon }B_{\varepsilon }d+D_{\varepsilon \chi }B_{\chi }d,\;\mu =D_{\chi \varepsilon }B_{\varepsilon }d+D_{\chi \chi }B_{\chi }d,$$

where

(15) $$\begin{array}{l} D_{\varepsilon \varepsilon }=\left[\begin{array}{cccc} A_{1111} & A_{1122} & A_{1112} & A_{1121}\\ A_{2211} & A_{2222} & A_{2212} & A_{2221}\\ A_{1211} & A_{1222} & A_{1212} & A_{1221}\\ A_{2111} & A_{2122} & A_{2112} & A_{2121} \end{array}\right],D_{\varepsilon \chi }=\left[\begin{array}{cc} B_{111} & B_{112}\\ B_{221} & B_{222}\\ B_{121} & B_{122}\\ B_{211} & B_{212} \end{array}\right],\\ D_{\chi \varepsilon }=\left[\begin{array}{cccc} C_{111} & C_{122} & C_{112} & C_{121}\\ C_{211} & C_{222} & C_{212} & C_{221} \end{array}\right],D_{\chi \chi }=\left[\begin{array}{cc} D_{11} & D_{12}\\ D_{21} & D_{22} \end{array}\right]. \end{array}$$

Note that, in the presence of centrosymmetric materials, the matrices $D_{\varepsilon \chi }$ and $D_{\chi \varepsilon }$, responsible for the coupling between classical and bending effects, turn to be zero as in the present work.

Finally, the discretized version of the weak statement governing the equilibrium problem of a micropolar body (neglecting also the body forces for the sake of simplicity) reads as $\delta d^{T}K\;d=\delta d^{T}F$ for any virtual displacement δd, where

(16) $$\begin{array}{l} K=\int_{\Omega }\left(B_{\varepsilon }^{T}D_{\varepsilon \varepsilon }B_{\varepsilon }+B_{\varepsilon }^{T}D_{\varepsilon \chi }B_{\chi }+B_{\chi }^{T}D_{\chi \varepsilon }B_{\varepsilon }+B_{\chi }^{T}D_{\chi \chi }B_{\chi }\right)d\Omega \\ F=\int_{\Gamma_{N}}\left[\begin{array}{c} N_{u}^{T}\;\bar{t}\\ N_{\phi }^{T}\;\bar{m} \end{array}\right]d\Gamma \end{array}$$

denote the stiffness matrix and the nodal force vector of the adopted finite element for describing a 2D linearly elastic and anisotropic micropolar body. Moreover, a standard Gauss integration technique is adopted for the computation of the stiffness terms appearing in Equation (16).

The above-described anisotropic Cosserat finite element was implemented within the COMSOL Multiphysics® environment, using the integrated Physics Builder functionality. Such a tool offers a user-friendly graphical interface for extending COMSOL’s built-in finite element library without requiring any in-depth programming skill [56].

### 3.2. Strong-Formulation Finite Element Method

Strong-form finite element method is a numerical approach based on a domain-decomposition technique merged with an extremely accurate pseudo-spectral collocation approach. As with any other strong-form methodology, both governing and boundary equations have to be implemented and solved at the same time. This leads to continuous stress and displacement fields across the element boundaries. In particular, the aforementioned pseudo-spectral approach considered here is the well-known generalized differential quadrature (GDQ) method. The GDQ method is able to approximate functional derivatives as a weighted linear sum of the functional values at a given regular grid of points. This required regularity limits the application of the GDQ to rectangular (or regular) domains only. Therefore, in order to study complex domains of arbitrary shape, the domain-decomposition technique (with mapping) has to be employed. Initially, the geometrical domain is divided into macro-elements according to the given geometrical or material discontinuities. Inside each element, a GDQ grid is placed according to a mapping transformation which might follow Lagrangian- or NURBS-based (Non-Uniform Rational Basis Spline) mapping [50,51,52]. In the present paper, the irregularity is due to a concentrated load which is applied on a linear edge; therefore, Lagrangian mapping with four nodes is sufficient to have an exact geometrical approximation. It was observed that the most common source of errors in the geometrical mapping is due to the presence of curvilinear boundaries [53,54]. In most of the published references, the GDQ method is presented in one-dimensional (1D) form because the numerical structure does not change much when the problem is 2D. In fact, for any given 2D function $f\left(\xi_{1},\xi_{2}\right)$, the following derivatives can be reported:

(17) $$\frac{\partial^{\left(n\right)}f\left(\xi_{1},\xi_{2}\right)}{\partial \xi_{1}^{\left(n\right)}}=c_{ik}^{\xi_{1}\left(n\right)}f_{kj},\;\frac{\partial^{\left(m\right)}f\left(\xi_{1},\xi_{2}\right)}{\partial \xi_{2}^{\left(m\right)}}=c_{jl}^{\xi_{2}\left(m\right)}f_{il},\;\frac{\partial^{\left(n+m\right)}f\left(\xi_{1},\xi_{2}\right)}{\partial \xi_{1}^{\left(n\right)}\partial \xi_{2}^{\left(m\right)}}=c_{ik}^{\xi_{1}\left(n\right)}\left(c_{jl}^{\xi_{2}\left(m\right)}f_{kl}\right),$$

where the repeated indices k and l indicate a sum up to the number of points $N_{\xi }$ for a fixed grid of $N_{\xi }\times N_{\xi }$, and the indices i and j indicate the point on the grid along the two directions $\xi_{1}$ and $\xi_{2}$, respectively. The indices n and m stands for the derivative order for the coordinate $\xi_{1}$ and $\xi_{2}$, respectively. It is remarked that the weighting coefficients $c_{ij}^{\xi_{1}},\;c_{ij}^{\xi_{2}}$ along $\xi_{1}$ and $\xi_{2}$ are different if a different number of points and collocation in the two directions are considered; otherwise, they are the same. Thus, for the sake of generality, Equation (17) is reported in general form, but the same number of points and grid collocation are considered in the numerical applications below.

Equation (17) has to be implemented into a computer code matrix form of the equations to be solved. Thus, Equation (17) can take the form below.

(18) $$C_{\xi_{1}}^{\left(n\right)}=c^{\xi_{1}\left(n\right)}⊗I,\;C_{\xi_{2}}^{\left(m\right)}=I⊗c^{\xi_{2}\left(m\right)},\;C_{\xi_{1}\xi_{2}}^{\left(n+m\right)}=c^{\xi_{1}\left(n\right)}⊗c^{\xi_{2}\left(m\right)},$$

where I is the identity matrix and $c^{\xi_{1}},c^{\xi_{2}}$ are the weighting coefficient matrices of Equation (17). Please note that the definitions in Equation (18) depend on how the collocation points are ordered in the computer code [52]. Equation (18) does not include the element mapping which modifies the algebraic equations according to selected procedure [52]; in fact, they are defined in the master (computational) element. Without losing generality, Equation (18) is mapped into Cartesian coordinates and can be presented as

(19) $$D_{x_{1}}^{\left(n\right)},\;D_{x_{2}}^{\left(m\right)},\;D_{x_{1}x_{2}}^{\left(n+m\right)}.$$

Now, Equation (19) can be used to carry out the discrete form of the governing equations as follows:

(20) $$\begin{array}{l} \left(A_{1111}D_{x_{1}}^{\left(2\right)}+2A_{1112}D_{x_{1}x_{2}}^{\left(2\right)}+A_{1212}D_{x_{2}}^{\left(2\right)}\right)U_{1}+\\ \left(A_{1121}D_{x_{1}}^{\left(2\right)}+\left(A_{1122}+A_{1221}\right)D_{x_{1}x_{2}}^{\left(2\right)}+A_{2212}D_{x_{2}}^{\left(2\right)}\right)U_{2}+\\ \left(B_{111}D_{x_{1}}^{\left(2\right)}+\left(B_{112}+B_{121}\right)D_{x_{1}x_{2}}^{\left(2\right)}+B_{122}D_{x_{2}}^{\left(2\right)}+\left(A_{1112}-A_{1121}\right)D_{x_{1}}^{\left(1\right)}+\left(A_{1212}-A_{1221}\right)D_{x_{2}}^{\left(1\right)}\right)\Omega +q_{1}=0 \end{array}$$

(21) $$\begin{array}{l} \left(A_{1121}D_{x_{1}}^{\left(2\right)}+\left(A_{1122}+A_{1221}\right)D_{x_{1}x_{2}}^{\left(2\right)}+A_{2212}D_{x_{2}}^{\left(2\right)}\right)U_{1}+\\ \left(A_{2121}D_{x_{1}}^{\left(2\right)}+2A_{2221}D_{x_{1}x_{2}}^{\left(2\right)}+A_{2222}D_{x_{2}}^{\left(2\right)}\right)U_{2}+\\ \left(B_{211}D_{x_{1}}^{\left(2\right)}+\left(B_{221}+B_{212}\right)D_{x_{1}x_{2}}^{\left(2\right)}+B_{222}D_{x_{2}}^{\left(2\right)}+\left(A_{1221}-A_{2121}\right)D_{x_{1}}^{\left(1\right)}+\left(A_{2212}-A_{2221}\right)D_{x_{2}}^{\left(1\right)}\right)\Omega +q_{2}=0 \end{array}$$

(22) $$\begin{array}{l} \left(B_{111}D_{x_{1}}^{\left(2\right)}+\left(B_{121}+B_{112}\right)D_{x_{1}x_{2}}^{\left(2\right)}+B_{122}D_{x_{2}}^{\left(2\right)}+\left(A_{1121}-A_{1112}\right)D_{x_{1}}^{\left(1\right)}+\left(A_{1221}-A_{1212}\right)D_{x_{2}}^{\left(1\right)}\right)U_{1}+\\ \left(B_{211}D_{x_{1}}^{\left(2\right)}+\left(B_{221}+B_{212}\right)D_{x_{1}x_{2}}^{\left(2\right)}+B_{222}D_{x_{2}}^{\left(2\right)}+\left(A_{2121}-A_{1221}\right)D_{x_{1}}^{\left(1\right)}+\left(A_{2221}-A_{2212}\right)D_{x_{2}}^{\left(1\right)}\right)U_{2}+\\ \left(D_{11}D_{x_{1}}^{\left(2\right)}+2D_{12}D_{x_{1}x_{2}}^{\left(2\right)}+D_{22}D_{x_{2}}^{\left(2\right)}+\left(2A_{1221}-A_{1212}-A_{2121}\right)\right)\Omega =0 \end{array}$$

The boundary conditions in terms of displacements are

(23) $$\begin{array}{l} U_{n}^{}=N_{1}U_{1}^{}+N_{2}U_{2}^{}={\bar{U}}_{N}\\ U_{t}^{}=N_{1}U_{1}^{}+N_{2}U_{2}^{}={\bar{U}}_{t}\\ \Omega =\bar{\Omega } \end{array}$$

where $N_{1}=n_{1}I,\;N_{2}=n_{2}I$. The boundary stresses in terms of displacements take the forms below.

(24) $$\begin{array}{l} \left(\left(A_{1111}D_{x_{1}}^{\left(1\right)}+A_{1112}D_{x_{2}}^{\left(1\right)}\right)N_{1}+\left(A_{1112}D_{x_{1}}^{\left(1\right)}+A_{1212}D_{x_{2}}^{\left(1\right)}\right)N_{2}\right)U_{1}+\\ \left(\left(A_{1121}D_{x_{1}}^{\left(1\right)}+A_{1122}D_{x_{2}}^{\left(1\right)}\right)N_{1}+\left(A_{1221}D_{x_{1}}^{\left(1\right)}+A_{2212}D_{x_{2}}^{\left(1\right)}\right)N_{2}\right)U_{2}+\\ \left(\left(B_{111}D_{x_{1}}^{\left(1\right)}+B_{112}D_{x_{2}}^{\left(1\right)}+A_{1112}-A_{1121}\right)N_{1}+\left(B_{121}D_{x_{1}}^{\left(1\right)}+B_{122}D_{x_{2}}^{\left(1\right)}+A_{1212}-A_{1221}\right)N_{2}\right)\Omega ={\bar{q}}_{1} \end{array};$$

(25) $$\begin{array}{l} \left(\left(A_{1121}D_{x_{1}}^{\left(1\right)}+A_{1221}D_{x_{2}}^{\left(1\right)}\right)N_{1}+\left(A_{1122}D_{x_{1}}^{\left(1\right)}+A_{2212}D_{x_{2}}^{\left(1\right)}\right)N_{2}\right)U_{1}+\\ \left(\left(A_{2121}D_{x_{1}}^{\left(1\right)}+A_{2221}D_{x_{2}}^{\left(1\right)}\right)N_{1}+\left(A_{2221}D_{x_{1}}^{\left(1\right)}+A_{2222}D_{x_{2}}^{\left(1\right)}\right)N_{2}\right)U_{2}+\\ \left(\left(B_{211}D_{x_{1}}^{\left(1\right)}+B_{212}D_{x_{2}}^{\left(1\right)}+A_{1221}-A_{2121}\right)N_{1}+\left(B_{221}D_{x_{1}}^{\left(1\right)}+B_{222}D_{x_{2}}^{\left(1\right)}+A_{2212}-A_{2221}\right)N_{2}\right)\Omega ={\bar{q}}_{2} \end{array};$$

(26) $$\begin{array}{l} \left(\left(B_{111}D_{x_{1}}^{\left(1\right)}+B_{121}D_{x_{2}}^{\left(1\right)}\right)N_{1}+\left(B_{112}D_{x_{1}}^{\left(1\right)}+B_{122}D_{x_{2}}^{\left(1\right)}\right)N_{2}\right)U_{1}+\\ \left(\left(B_{211}D_{x_{1}}^{\left(1\right)}+B_{221}D_{x_{2}}^{\left(1\right)}\right)N_{1}+\left(B_{212}D_{x_{1}}^{\left(1\right)}+B_{222}D_{x_{2}}^{\left(1\right)}\right)N_{2}\right)U_{2}+\\ \left(\left(D_{11}D_{x_{1}}^{\left(1\right)}+D_{12}D_{x_{2}}^{\left(1\right)}+B_{121}-B_{221}\right)N_{1}+\left(D_{12}D_{x_{1}}^{\left(1\right)}+D_{22}D_{x_{2}}^{\left(1\right)}+B_{122}-B_{212}\right)N_{2}\right)\Omega =0 \end{array}.$$

The complete set of governing (Equations (20)–(22)) and boundary (Equations (24)–(26)) equations can be collected (after re-ordering) into the following algebraic system reported in matrix form KU+Q=0 by separating boundary and domain grid points as

(27) $$K=\left[\begin{array}{cc} K_{bb} & K_{bd}\\ K_{db} & K_{dd} \end{array}\right],\;U=\left[\begin{array}{c} U_{b}\\ U_{d} \end{array}\right],\;Q=\left[\begin{array}{c} Q_{b}\\ Q_{d} \end{array}\right],$$

where $K_{bb},\;K_{bd}$ indicate the discrete form of the boundary equations, $K_{db},\;K_{dd}$ represent the matrices of the fundamental equations, $Q_{b}$, $Q_{d}$ are the vectors of boundary and domain forces, and $U_{b},\;U_{d}$ are the rearranged vectors of the displacement parameters ($U_{1},\;U_{2},\;\Omega$) divided into boundary and domain degrees of freedom. Finally, to improve the numerical stability, the static condensation is performed so that

(28) $$U_{d}={\left(K_{dd}-K_{db}K_{bb}^{-1}K_{bd}\right)}^{-1}\left(K_{db}K_{bb}^{-1}Q_{b}-Q_{d}\right)\;\to \;\hat{U}={\hat{K}}^{-1}\hat{Q},$$

where

(29) $$U_{b}=-K_{bb}^{-1}\left(F_{b}+K_{bd}U_{d}\right).$$

Clearly, Equation (28) can be solved using any numerical tool using, for instance, Cholesky decomposition as used by MATLAB. It is from Equation (23) that the total number of degrees of freedom (DOFs) in each problem for the SFEM can be computed as $3n_{e}{\left(N_{\xi }-2\right)}^{2}$, where 3 is the number of DOFs per grid point, $n_{e}$ is the number of elements in the mesh, and ${\left(N_{\xi }-2\right)}^{2}$ is the total number of grid points per element.

## 4. Numerical Simulations

**Figure 1:**
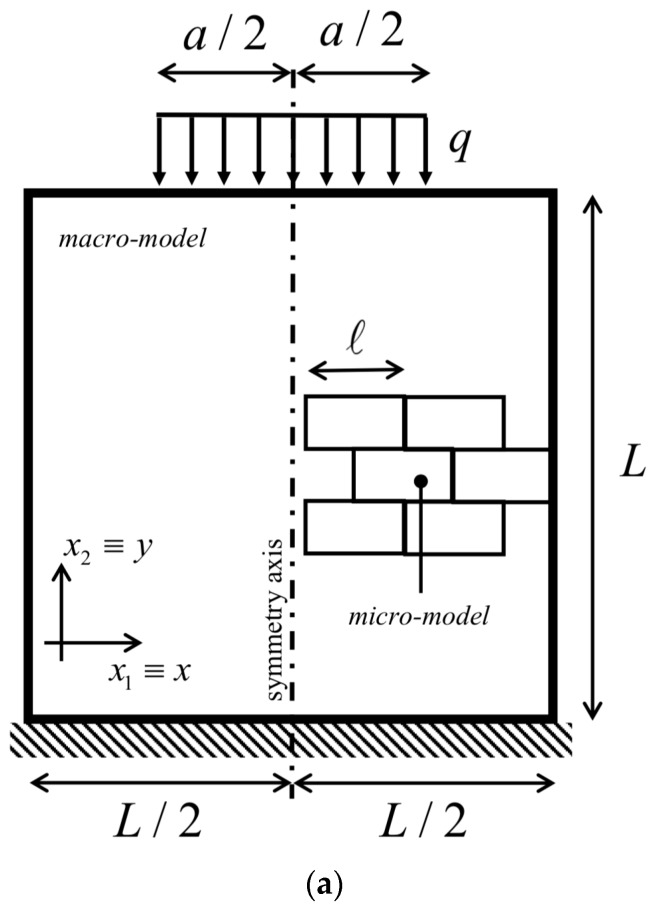

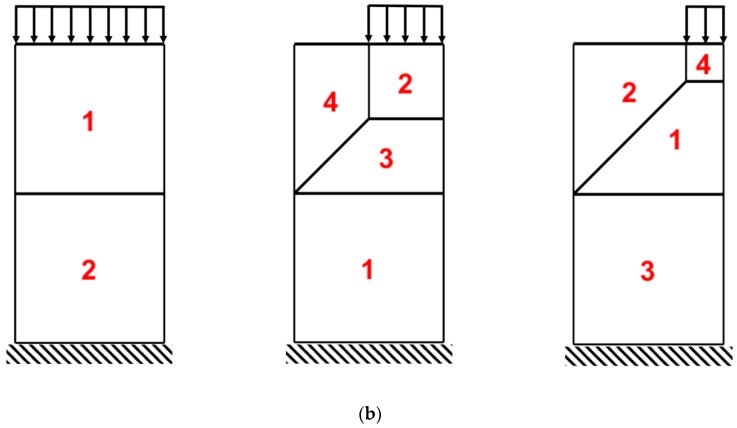
Geometric configurations: (**a**) general sketch of loading and boundary conditions of the wall considered for numerical simulations; (**b**) detail of each panel wall with clamped and top load conditions: Case 1 ($a_{1}/L=1$), Case 2 ($a_{2}/L=0.5$), and Case 3 ($a_{3}/L=0.25$).

**Figure 2:**
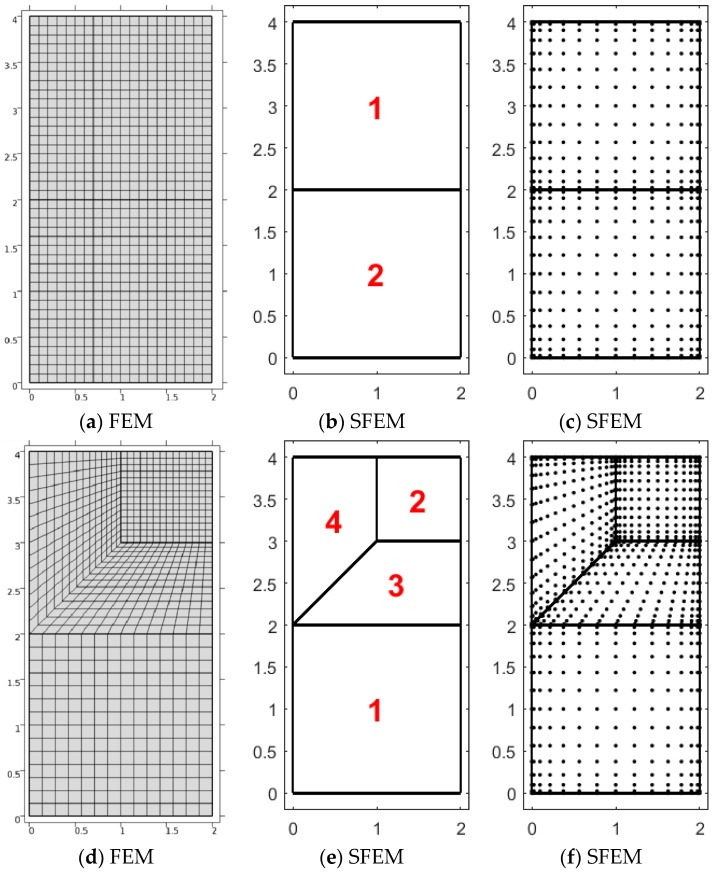

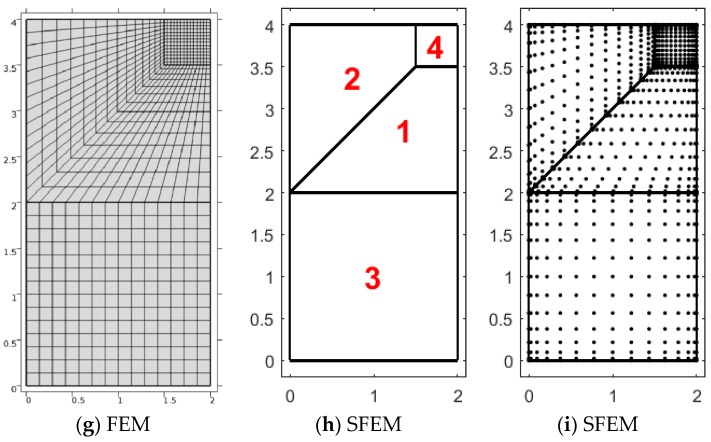
Numerical discretizations for both finite element model (FEM) and strong-form finite element model (SFEM): (**a**–**c**) Case 1 ($a_{1}/L=1$), (**d**–**f**) Case 2 ($a_{2}/L=0.5$), and (**g**–**i**) Case 3 ($a_{3}/L=0.25$). Finite element mesh for the FEM, macro-element discretization, and mesh-free points for the SFEM are shown.

The present study aimed to compare the two different numerical approaches adopted in the modeling of orthotropic micropolar continua. The problem illustrated below considers a square domain/wall of width/side L=4 m, fixed at the bottom edge and subjected to several top loads acting on lengths of different size a according to three ratios $a_{1}/L=1$, $a_{2}/L=0.5$, and $a_{3}/L=0.25$. A general sketch of the present geometry is depicted in Figure 1a,b, representing the three half-wall geometries termed Case 1, Case 2, and Case 3 used in the computations with evidence of the top load and bottom boundary condition used. For the sake of comparison, the resultant of the top load is kept constant for all three cases above as F=10 MN. Thus, the intensities of the equivalent distributed force for the three geometrical configurations are $q_{1}=2.5\;\mathrm{MPa}$, $q_{2}=5\;\mathrm{MPa}$, and $q_{3}=10\;\mathrm{MPa}$, respectively. It is remarked that the physical problem is studied for the three different configurations (Case 1, Case 2, and Case 3) for different values of the load size footprint $a_{1}$, $a_{2}$, and $a_{3}$, associated with decreasing value of the ratio a/L, which is responsible for the “structural size effects”. As such a ratio tends to a value much smaller than 1, the considered distributed load behaves as a concentrated top vertical force F acting at the symmetry line of the panel. The load sketches considered in Figure 1b imply the mesh patterns selected as reported in Figure 2, not for numerical convergence reasons, but for capturing the load discontinuity given.

In order to have a comparison, the medium was modeled as a classical and micropolar equivalent continuum. The adopted material constants are summarized in Table 1 and were evaluated using the coarse-graining approach presented in Reference [26]. With ℓ as the significant microstructure internal length of the composite assembly, dependent on the brick size, a scale ratio ℓ/L governs the so-called “material size effects” of the considered panel. Three material cases corresponding to running bond sequences of bricks of increasing size were analyzed, named Material 1 ($\ell_{1}/L=0.005$), Material 2 ($\ell_{2}/L=0.05$), and Material 3 ($\ell_{3}/L=0.5$) [26]. Note that, as the Cauchy model does not depend on the size, the corresponding constitutive parameters do not vary for these three material cases. Moreover, the variation of the brick size does not affect the independent micropolar constants A1111, A1122, A1212, and A2121, while the bending moduli, D11 and D12, are strongly affected and play a fundamental role in retaining memory of the original discrete microstructure. In all the effected simulations, for the sake of simplicity, the out-of-diagonal terms are set equal to zero, and this corresponds to neglect dilatant effects in the joints between the bricks of the 2D composite solid considered.

The final aim was to show the capability of the micropolar model to retain memory of the original composite behavior under the action of a load applied on a limited area, as well as to numerically investigate the related mechanism of diffusion, using both FEM and SFEM approaches.

Due to the symmetry of the problem, only half of the domain was numerically studied, and the discretizations correspond to the aforementioned geometries (Figure 2) for both FEM and SFEM. The finite element discretization is based on the macro-element decomposition. For the SFEM, two representations are provided: the one with macro-elements (domain decomposition) and the other with both elements and grid points. With this discretization, it was easier to determine a closer match in terms of degrees of freedom between FEM and SFEM. It can be noted that, in Case 1, the load is uniform ($a_{1}/L=1$); thus, only two elements are needed for SFEM. Actually, SFEM could handle it with just one element, but that would lead to a larger mesh distortion in the FEM counterpart. On the contrary, the other two problems, Case 2 ($a_{2}/L=0.5$) and Case 3 ($a_{3}/L=0.25$), consider four elements with the load applied on the top-right element of the given macro-element mesh pointing downward.

**Figure 3:**
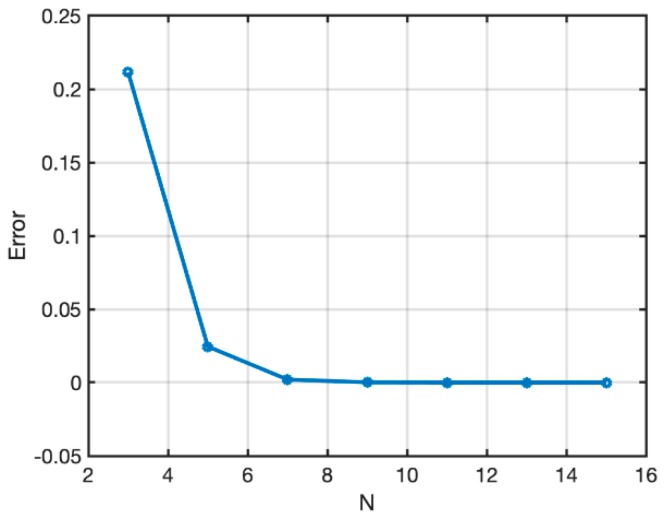
Convergence analysis of the SFEM for Case 3 ($a_{3}/L=0.25$) and Material 1 ($\ell_{1}/L=0.005$) by varying the number of points N inside each macro-element in the given mesh.

In order to show the numerical stability of the present SFEM numerical approach, convergence analysis of Case 3 is presented with material configuration Material 1 ($\ell_{1}/L=0.005$) as indicated in Table 1. A Chebyshev–Gauss–Lobatto grid [49] was considered by varying the number of grid points inside each element. The vertical displacement of the point on the symmetry axis of the geometry Case 3 under vertical pressure was considered as a reference value. The “error” between the displacement, computed by varying the number of points and the reference one with 15×15 points, was considered and is plotted in Figure 3. A negligible “error” was measured for such a 15×15 mesh, but it is clear that, for a lower number of points, larger differences emerge. Evidence of the numerical stability is clear from Figure 3, where it is clearly shown that the numerical technique becomes stable for 15×15 points. Therefore, such a selection was considered thereafter. Nevertheless, the presentation of the detailed numerical accuracy of both FEM and SFEM is out of the scope of the present study; thus, it is only marginally presented. In the authors’ previous work [48], it was already shown that SFEM reaches numerical stability prior to FEM due to its strong-form nature. Moreover, stress plots in SFEM are continuous among elements; instead, FEMs have stress jumps because stresses are post-computed in the integration (interior) points of the element and not enforced a priori as in SFEM. For letting FEM contour maps be continuous as in SFEM, stress interpolation was applied among elements. It was observed in the literature that Chebyshev–Gauss–Lobatto grid [49] distribution provides the most accurate results and a uniform convergence by increasing the number of grid points. FEM models have uniformly distributed mesh elements, in particular, 21×21 for Case 1, and 15×15 for Case 2 and Case 3 for each macro-element. According to the elements used and by considering that FEM models are made of nine-node elements, the number of DOFs for Case 1 is 1014 for SFEM and 7503 for FEM, and the number of DOFs for Case 2 and Case 3 is 2028 for SFEM and 7339 for FEM.

The results are presented in terms of the two continuum models (classical or micropolar), for the three geometries (Figure 1b) and material cases considered (Table 1). Therefore, a total of 12 configurations are provided. It can be noted that the bending moduli, responsible of the scale effects, also indirectly affect the relative rotations between the local rigid rotation, i.e., the macro-rotation $\theta =0.5\left(u_{2,1}-u_{1,2}\right)$, and the micro-rotation, ϕ, which is associated with non-symmetric angular strain components; therefore, they also have influence on the anisotropic features of the constitutive relations. Therefore, even if we did not consider a direct variation in the material symmetry (orthotropy) of the microstructure, the scale effect indirectly affects the results in terms of relative rotation. Conversely, the shear parameters, $A_{1212}$ and $A_{2121}$, are directly associated with the resulting micropolar orthotropy of the panel, meaning that their values significantly influence the relative rotations. The direct variation in the material orthotropy is the object of a future work.

**Figure 4:**
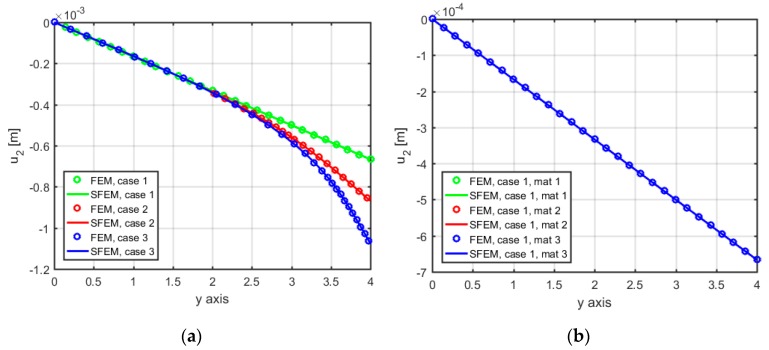

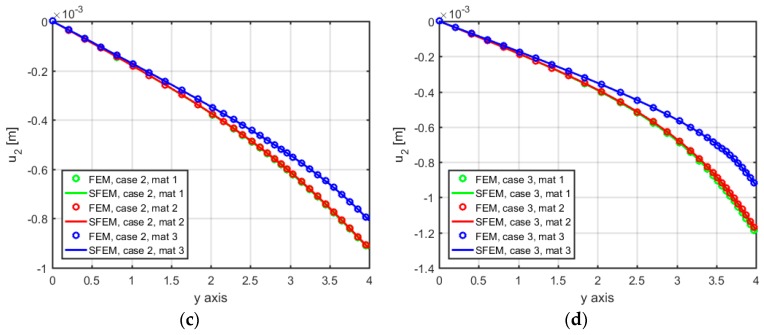
Displacement component $u_{2}$ along the symmetry axis: (**a**) Cauchy; (**b**) Case 1 ($a_{1}/L=1$); (**c**) Case 2 ($a_{2}/L=0.5$); (**d**) Case 3 ($a_{3}/L=0.25$).

Figure 4 shows the comparison between the two numerical approaches in terms of the vertical displacement, $u_{2}$, along the symmetry axis of the problem. Figure 4a represents the classical material configuration, together with the three geometrical cases, whereas Figure 4b–d report each case separately by varying the micropolar material in each figure. From Figure 4a, it can be noted that a reduction of the load footprint size leads to a local stress concentration (as deduced from the nonlinear behavior of the vertical displacements for Cases 2 and 3), which is absent in Case 1 (characterized by a uniform vertical displacement state). FEM solutions are indicated with solid lines and SFEM ones are indicated with markers. It is evident that the two solutions accurately match; therefore. it can be said that both numerical tools are able to describe these phenomena. As expected, Case 1 ($a_{1}/L=1$) (Figure 4b) has a uniform top load and a uniform fixed boundary at the bottom and gives the same results for any (Cauchy or micropolar) material configuration, highlighting that the micropolar model shows different solutions from the classical elastic approach only in the presence of force concentrations, whereby it is able to activate rotational DOFs and also produce relative rotations between macro- and micro-rotations. In other words, Case 1, without any stress concentration, is not sensitive to the “scale effect” due to a change in the material constants $D_{11}$ and $D_{22}$, and the results for Cosserat and Cauchy materials are coincident in terms of displacements and stresses.

On the contrary, Case 2 and Case 3, reported in Figure 4c,d, respectively, differ among themselves as a function of the configuration (classical or micropolar) and material properties (Material 1 ($\ell_{1}/L=0.005$), Material 2 ($\ell_{2}/L=0.05$), and Material 3 ($\ell_{3}/L=0.5$)). The effect due to the applied load is obvious: concentrated loads ($a_{2}/L=0.5$ for Case 2 and $a_{3}/L=0.25$ for Case 3) give larger displacements, whereas the material configurations give different results if Case 2 and Case 3 are observed. In particular, the increase of the internal material length, through $D_{11}$ and $D_{22}$, which is an intrinsic feature of the micropolar constitutive behavior, alleviates the stress concentration at the top, thus reducing the vertical displacements with respect to the classical case of Figure 4a.

**Figure 5:**
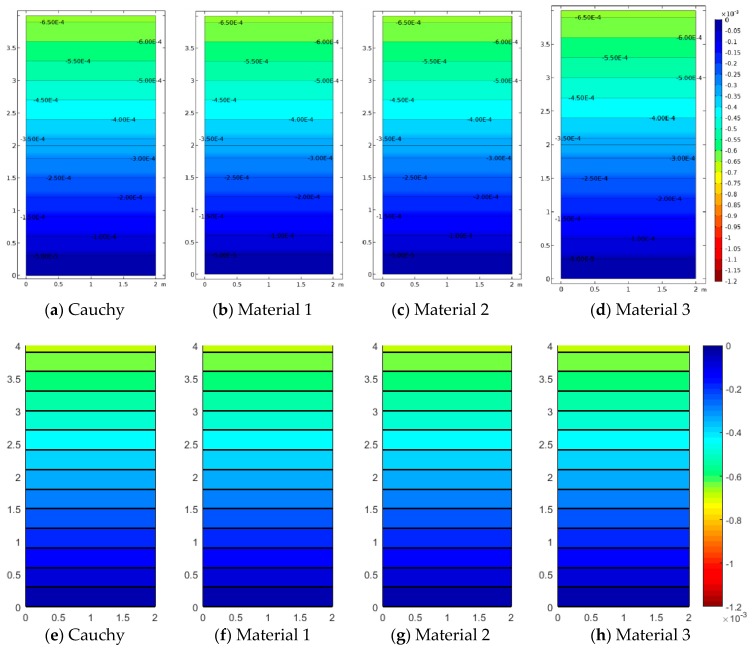
Case 1 ($a_{1}/L=1$): $u_{2}$ contour plots for Cauchy, Material 1 ($\ell_{1}/L=0.005$), Material 2 ($\ell_{2}/L=0.05$), and Material 3 ($\ell_{3}/L=0.5$; (**a**–**d**) FEM, (**e**–**h**) SFEM.

**Figure 6:**
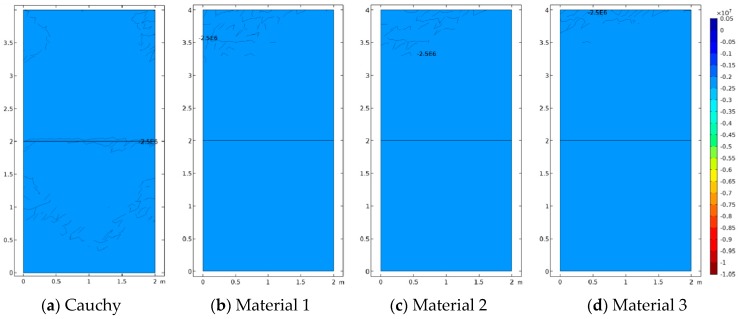

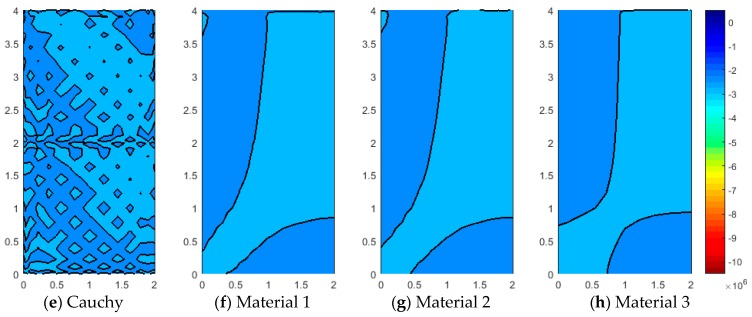
Case 1 ($a_{1}/L=1$) vertical stress $\sigma_{22}$ contour plots for Cauchy, Material 1 ($\ell_{1}/L=0.005$), Material 2 ($\ell_{2}/L=0.05$), and Material 3 ($\ell_{3}/L=0.5$; (**a**–**d**) FEM, (**e**–**h**) SFEM.

**Figure 7:**
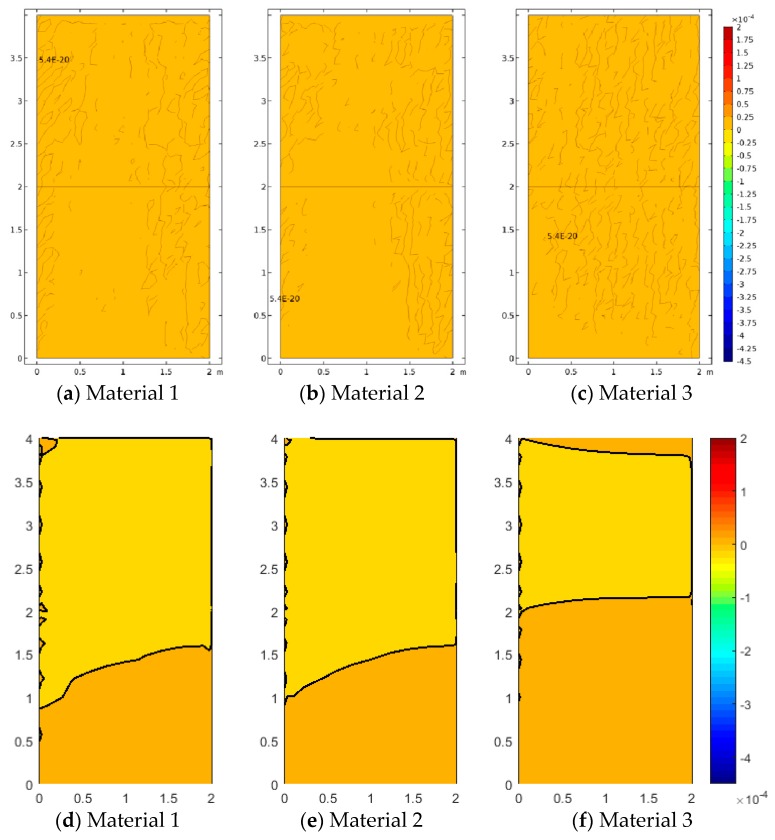
Case 1 ($a_{1}/L=1$) relative rotation θ−ϕ for Material 1 ($\ell_{1}/L=0.005$), Material 2 ($\ell_{2}/L=0.05$), and Material 3 ($\ell_{3}/L=0.5$; (**a**–**d**) FEM, (**e**–**h**) SFEM.

Figure 5, Figure 6 and Figure 7 represent the vertical displacement $u_{2}$, vertical stress $\sigma_{22}$, and relative rotation, $\theta -\phi =0.5\left(u_{2,1}-u_{1,2}\right)-\phi$, for Case 1 using both FEM and SFEM. This case has a uniform top load and a uniform clamped boundary condition at the bottom so that no load or geometrical discontinuity is present. Therefore, as expected and already observed above (Figure 4b), there is no difference among classical and micropolar solutions. The given results, for all cases, show a linear displacement field (Figure 5) with maximum values at the top where the pressure load is applied and zero values at the bottom where the boundary condition is enforced. Due to the particular boundary and loading conditions for the present case, the vertical stress field $\sigma_{22}$ is constant in the whole domain (Figure 6). Please note that, due to very small numerical oscillations, the color map in the representation is not of a single color but two colors, or else the same scattered contour lines might appear. This does not change the fact that the whole stress map is of a constant value $\sigma_{22}=-2.5\;\mathrm{MPa}$ as indicated. Finally, similar comments can be reported for Figure 7, where relative rotation, θ−ϕ, is shown. The relative rotation for Case 1 is zero, as expected, because there is no micropolar effect in structures without (geometric or material) discontinuities. It is also evident from these plots that not only are classical and micropolar cases equal but so are the solutions provided by FEM and SFEM, in terms of contour plots. The relative rotation is not shown for the classical model because, for such a case, the micropolar rotation ϕ is not present, as only the macro-rotation can be computed in the Cauchy model.

**Figure 8:**
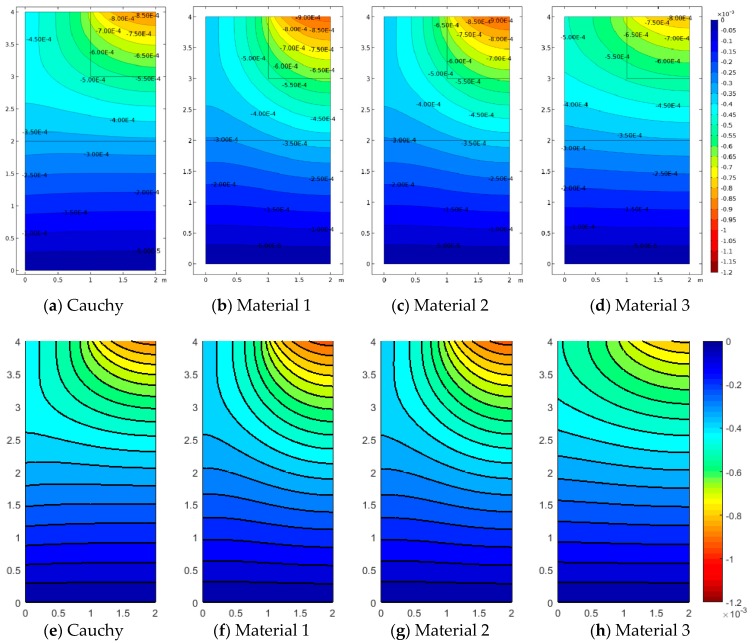
Case 2 ($a_{2}/L=0.5$): $u_{2}$ contour plots for Cauchy, Material 1 ($\ell_{1}/L=0.005$), Material 2 ($\ell_{2}/L=0.05$), and Material 3 ($\ell_{3}/L=0.5$; (**a**–**d**) FEM, (**e**–**h**) SFEM.

**Figure 9:**
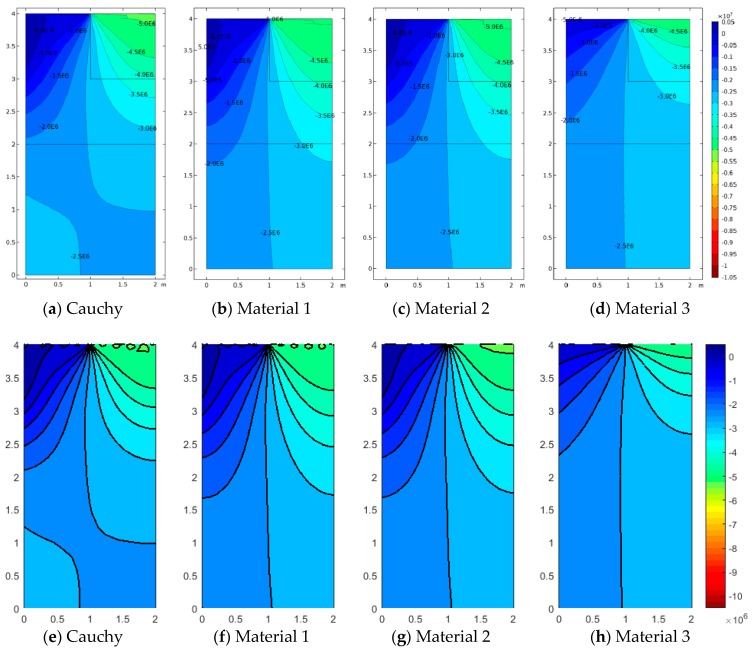
Case 2 ($a_{2}/L=0.5$) vertical stress $\sigma_{22}$ contour plots for Cauchy, Material 1 ($\ell_{1}/L=0.005$), Material 2 ($\ell_{2}/L=0.05$), and Material 3 ($\ell_{3}/L=0.5$; (**a**–**d**) FEM, (**e**–**h**) SFEM.

**Figure 10:**
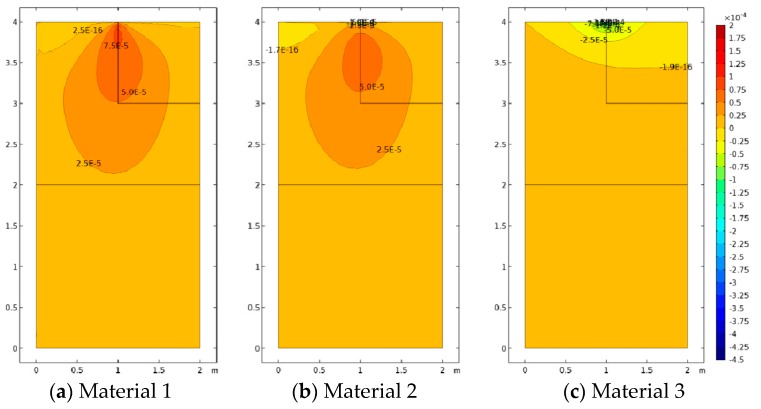

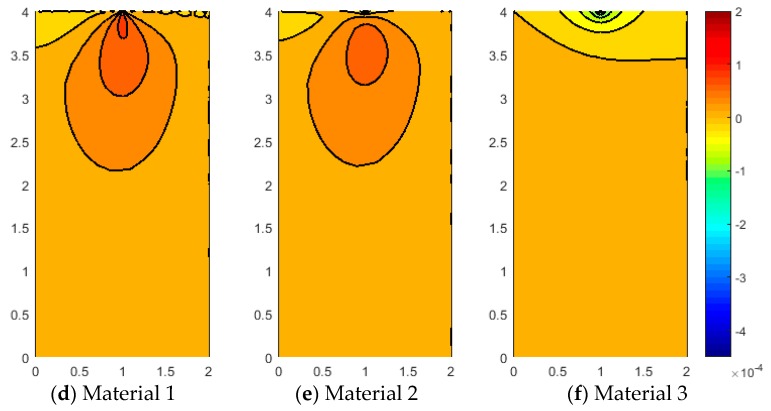
Case 2 ($a_{1}/L=0.5$) relative rotation θ−ϕ for Material 1 ($\ell_{1}/L=0.005$), Material 2 ($\ell_{2}/L=0.05$), and Material 3 ($\ell_{3}/L=0.5$; (**a**–**d**) FEM, (**e**–**h**) SFEM.

In Case 2 and Case 3, the effect of concentrated loads and difference materials is shown. Case 2 is depicted in Figure 8, Figure 9 and Figure 10. For all cases, FEM and SFEM also agree very well. It should be pointed out that, in terms of displacements for the Cauchy case (Figure 8a), the map has contour lines which tend to lay horizontally on the whole medium except for the zone directly influenced by the load, whereas micropolar materials (Figure 8b–d and Figure 8f–h) have contour lines which are wider-spread in the domain. It is remarked that Material 1 ($\ell_{1}/L=0.005$) has higher displacements close to the area where the load is applied and almost zero displacement near the free boundary (left boundary), whereas Material 3 ($\ell_{3}/L=0.5$) has the lower displacement value with strongly not-zero displacement on the free boundary, in agreement with the results in Figure 4c,d and Figure 4g,h. In fact, Material 3 is the one with the higher micropolar effect, due to higher values of the $D_{11}$ and $D_{22}$ parameters, resulting in the significant reduction of strain (and stress) gradients. The contour plot solutions in terms of stress $\sigma_{22}$ (Figure 9) reflect the aforementioned results in terms of the displacement components. In fact, the highest stress contour map is given by the classical configuration, and the micropolar Material 3 has the lowest stress field with the small stress distribution within the medium. In other words, Material 3 is associated with the most significant reduction of stress (and strain) concentrations. The micropolar material models are able to re-distribute the stresses within elastic media better than classical elasticity [48]. This effect is tailored by the micropolar mechanical properties and is stronger when $D_{11}$ and $D_{22}$ have higher values, which are associated with larger ratios, where ℓ/L is the material intrinsic length. The micropolar effect can be easily observed from the relative rotation contour plots (Figure 10). In fact, all micropolar materials show a strong effect between the area where the load is applied and the free surface at the top boundary; in order words, the relative rotation has high values close to the discontinuity. Such an effect is much stronger for the micropolar solid with the highest bending constants (Material 3) with respect to the others. However, Material 1 and Material 2 show a relative rotation that goes down toward the boundary edge, whereas Material 3 has a relative rotation closer to the top free surface.

**Figure 11:**
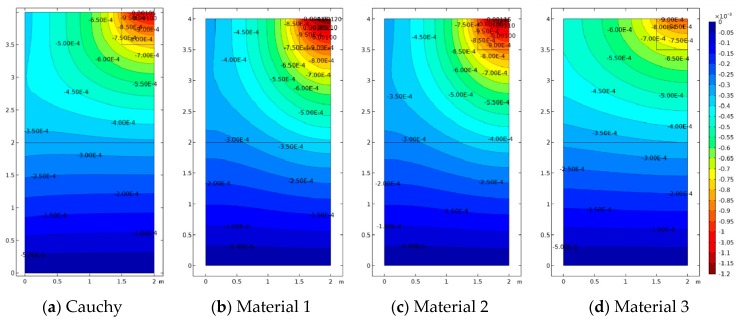

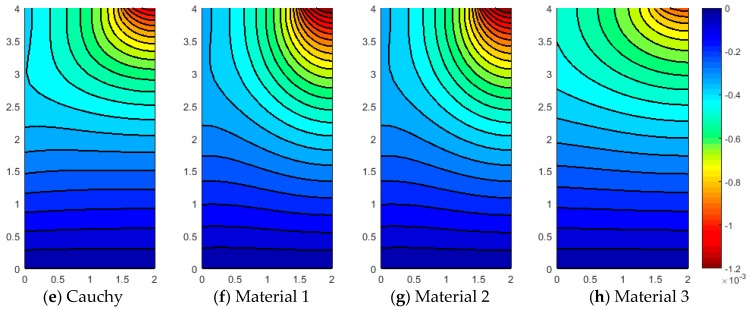
Case 3 ($a_{3}/L=0.25$): $u_{2}$ contour plots for Cauchy, Material 1 ($\ell_{1}/L=0.005$), Material 2 ($\ell_{2}/L=0.05$), and Material 3 ($\ell_{3}/L=0.5$; (**a**–**d**) FEM, (**e**–**h**) SFEM.

**Figure 12:**
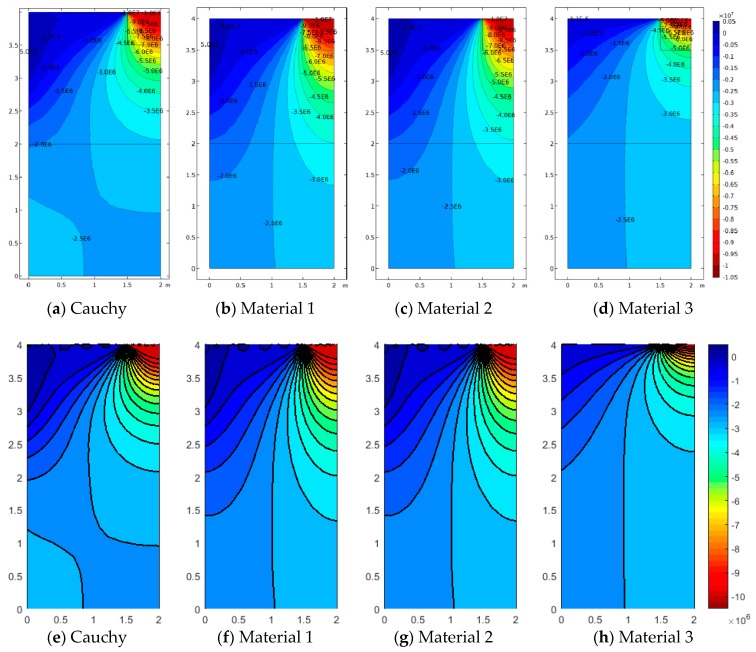
Case 3 ($a_{3}/L=0.25$) vertical stress $\sigma_{22}$ contour plots for Cauchy, Material 1 ($\ell_{1}/L=0.005$), Material 2 ($\ell_{2}/L=0.05$), and Material 3 ($\ell_{3}/L=0.5$; (**a**–**d**) FEM, (**e**–**h**) SFEM.

**Figure 13:**
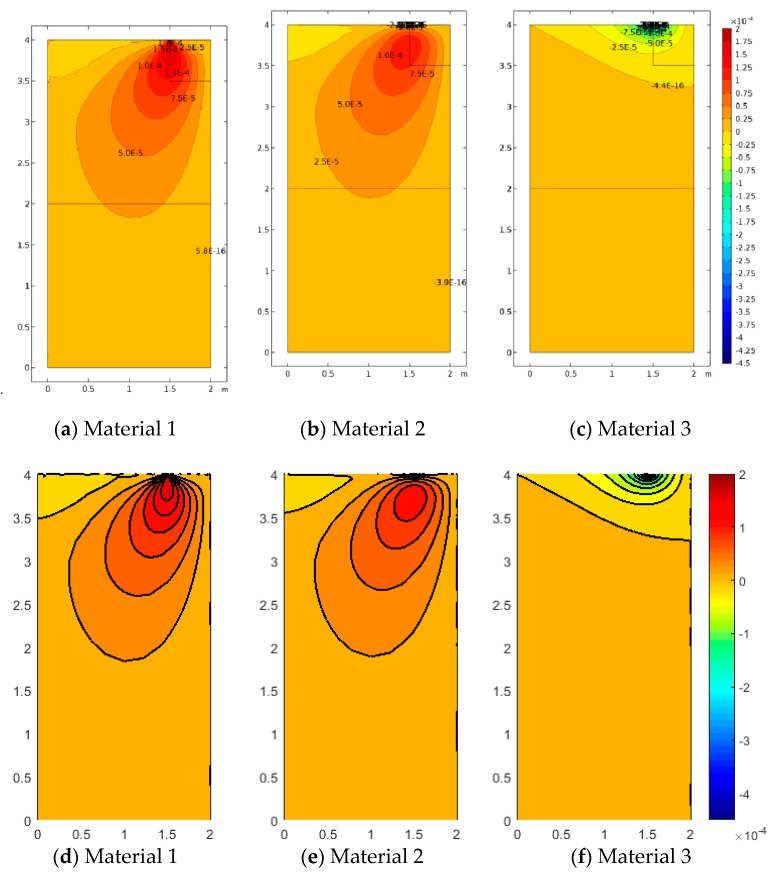
Case 3 ($a_{3}/L=0.25$) relative rotation θ−ϕ for Material 1 ($\ell_{1}/L=0.005$), Material 2 ($\ell_{2}/L=0.05$), and Material 3 ($\ell_{3}/L=0.5$; (**a**–**d**) FEM, (**e**–**h**) SFEM.

Analogous comments can be reported for Case 3 ($a_{3}/L=0.25$), where the concentrated load is much stronger (applied on a smaller area) in Figure 11, Figure 12 and Figure 13. Obviously, the corresponding values of vertical displacements $u_{2}$, vertical stresses $\sigma_{22}$ and relative rotations,θ−ϕ, are higher than the previous cases, even though the resultant force has equal magnitude. This is due to the fact that the geometrical discontinuity is stronger in Case 3 (Figure 4). Moreover, it can be observed that the gradients represented in Figure 11, Figure 12 and Figure 13 are stronger with respect to Figure 8, Figure 9 and Figure 10, as confirmed by the reported contour lines. Figure 13 in particular shows that Material 1 (associated with the smallest scale ratio $\ell_{1}/L=0.005$) presents the most remarkable anisotropic features, providing the greatest peak for the relative rotations (related to the antisymmetric part of the strain tensor), as well as for the strain (curvature) and stress components. In this case, the load diffusion is less evident, and the vertical deflection is larger than in the other considered cases, thus highlighting smaller overall flexural stiffness properties. Another observation can be made on Figure 13, related to the relative rotation, whereby contour lines are slightly rotated with respect to Figure 10. This is due to the fact that the geometrical discontinuity in Case 3 is not aligned with the center of the half-medium presented here as in Case 2. In fact, in both cases, the relative rotation for Material 1 and Material 2 points toward the center of the medium; however, since in Case 2 the discontinuity is already aligned with the center, the contour plot shape is almost symmetric, whereas, for Case 3, the latter is slightly deformed.

The results here obtained are in agreement with the results already presented in previous works [20,26] and the extended simulations contained herein. The comparison with new experimental results will be the object of a future work.

## 5. Conclusions

This work proposed a detailed numerical investigation of the scale effects in orthotropic composites, such as brick/block assemblies, modeled as micropolar continua, under the action of localized loads. The numerical solution of the underlying boundary value problems was performed using two different numerical methods, i.e., the finite element method and the strong-formulation finite element method (SFEM). The results show that FEM and SFEM approaches provide comparable results both for classical and micropolar materials. The effect of orthotropic micropolar mechanical properties was shown as a function of geometrical discontinuities and the material properties, derived from the description of the composite microstructure. In particular, two length scales were considered in the present study, i.e., the scale ratio a/L between the characteristic size a of the loaded area and the overall structural size, chosen as a suitable measure of the load concentration, and the scale ratio ℓ/L, with ℓ being the internal material length that directly affects the scale-dependent micropolar bending moduli.

It was observed that the relative rotation represents a significant measure of the micropolar effect of an elastic medium subjected to concentrated loads. For the trivial solution with constant top pressure (i.e., fixing $a_{1}/L=1$), no micropolar effect was also carried out and shown. In fact, for such a configuration, no difference could be observed between classical and micropolar models. By tailoring the micropolar mechanical properties. the effect on orthotropic media changes both in terms of displacements and stresses. With reference to the cases of nonhomogeneous stress states (i.e., Case 2 $a_{2}/L=0.5$, and Case 3 $a_{3}/L=0.25$) associated with localized loads (similarly to the case of other geometry or boundary discontinuities), it was shown that the contribution of the relative rotation between the macro- and the micro-rotation can be increased by using higher values of the micropolar bending moduli corresponding to increasing values of the scale ratios, ℓ/L. Its beneficial role allows the diffusion of concentrated loads, thus alleviating the associated severe stress gradients, especially for Case 3 (i.e., that associated with the smallest ratio $a_{3}/L=0.25$), thus highlighting the capability of the micropolar model to retain memory of the underlying microstructure response under the action of localized loads.

Moreover, the micropolar bending moduli were proven to be influenced by the anisotropic features of the constitutive relations, which directly affect the micropolar shear parameters, kept fixed in the present study. In the cases of nonhomogeneous stress states under investigation, Material 1 (associated with the smallest scale ratio $\ell_{1}/L=0.005$) showed the most remarkable anisotropic features. In this case, the load diffusion was less evident, the vertical deflection was larger than in the other considered cases, and the relative rotations (related to the antisymmetric part of the strain tensor) showed the greatest peak, thus highlighting smaller overall flexural stiffness properties.

From a numerical point of view, it was also shown that, even if in the cases analyzed the two solutions provide the same results, SFEM is a more reliable and simple technique to apply in terms of discretization procedure, because the mesh can be constructed starting from the discontinuities present in the physical problem. Many finite elements have to be used in all computations, and mesh refinements have to be applied for refining the numerical solutions. On the contrary, once the mesh is defined in terms of macro-elements in SFEM, a simple re-population in terms of grid collocation points can be easily performed. Moreover, SFEM needs only to employ derivative discretization, whereas FEM implements both integrals and derivatives, which leads to slower calculations in terms of computational cost.

## Figures and Tables

**Table 1:** Mechanical properties used in all the computations.

Cauchy Model	Micropolar Model	
$\begin{array}{l} {\hat{A}}_{1111}=A_{1111}=3.75\cdot 10^{4}\mathrm{MPa}\\ {\hat{A}}_{2222}=A_{2222}=1.5\cdot 10^{4}\mathrm{MPa}\\ {\hat{A}}_{1212}=\left(A_{1212}+A_{2121}\right)/2=1.875\cdot 10^{4}\mathrm{MPa} \end{array}$	$\begin{array}{l} A_{1111}=3.75\cdot 10^{4}\mathrm{MPa}\\ A_{2222}=1.5\cdot 10^{4}\mathrm{MPa}\\ A_{1212}=0.75\cdot 10^{4}\mathrm{MPa}\\ A_{2121}=3\cdot 10^{4}\mathrm{MPa} \end{array}$	
	Material 1 (block size 0.02×0.01, $\ell_{1}/L$ [26])	$\begin{array}{l} D_{11}=1.125\;\mathrm{MN}\\ D_{22}=0.375\;\mathrm{MN} \end{array}$
	Material 2 (block size 0.20×0.10, $\ell_{2}/L$ [26])	$\begin{array}{l} D_{11}=112.5\;\mathrm{MN}\\ D_{22}=37.5\;\mathrm{MN} \end{array}$
	Material 3 (block size 2.00×1.00, $\ell_{3}/L$ [26])	$\begin{array}{l} D_{11}=11250\;\mathrm{MN}\\ D_{22}=3750\;\mathrm{MN} \end{array}$
$A_{1112}=A_{1121}=A_{2212}=A_{2221}=A_{1122}=A_{1221}=0$, $B_{111}=B_{112}=B_{221}=B_{222}=B_{121}=B_{122}=B_{211}=B_{212}=0$, $D_{12}=0$		
